# Results of postoperative radiochemotherapy of the patients with resectable gastroesophageal junction adenocarcinoma in Slovenia

**DOI:** 10.2478/v10019-012-0049-4

**Published:** 2012-11-09

**Authors:** Ana Jeromen, Irena Oblak, Franc Anderluh, Vaneja Velenik, Marija Skoblar Vidmar, Ivica Ratoša

**Affiliations:** Department of Radiotherapy, Institute of Oncology Ljubljana, Slovenia

**Keywords:** gastroesophageal junction adenocarcinoma, postoperative radiochemotherapy, toxicity

## Abstract

**Background.:**

Although the incidence of adenocarcinomas of the gastroesophageal junction (GEJ) is sharply rising in the Western world, there are still some disagreements about the staging and the treatment of this disease. The aim of this retrospective study was to analyse the effectiveness and safety of postoperative radiochemotherapy in patients with a GEJ adenocarcinoma treated at the Institute of Oncology Ljubljana.

**Patients and methods.:**

Seventy patients with GEJ adenocarcinoma, who were treated with postoperative radiochemotherapy between January 2005 and June 2010, were included in the study. The treatment consisted of 6 cycles of chemotherapy with 5-FU and cisplatin and concomitant radiotherapy with the total dose of 45 Gy.

**Results.:**

Twenty-six patients (37.1%) completed the treatment according to the protocol. The median follow-up time was 17.7 months (range: 3.3–64 months). Acute toxicity grade 3 or more, such as stomatitis, dysphagia, nausea or vomiting, and infection, occurred in 2.9%, 34.3%, 38.6% and 41.5% of patients, respectively. At 3 years locoregional control (LRC), disease-free survival (DFS), disease-specific survival (DSS) and overall survival (OS) were 78.2%, 25.3%, 35.8%, and 33.9%, respectively. In the multivariate analysis of survival, splenectomy and level of Ca 19-9 >20 kU/L before the adjuvant treatment were identified as independent prognostic factors for lower DFS, DSS and OS. Age <60 years, higher number of involved lymph nodes and advanced disease stage were identified as independent prognostic factors for lower DSS and OS.

**Conclusions.:**

In patients with GEJ adenocarcinoma who first underwent surgery, postoperative radiochemotherapy is feasible, but we must be aware of a high risk of acute toxic side effects.

## Introduction

Adenocarcinomas of the gastroesophageal junction (GEJ) represent a heterogeneous group of tumours with poor prognosis. They are defined as tumours, which arise within 5 cm proximal or distal to the esophagogastric junction.^1^ Despite a dramatic rise in the incidence of GEJ adenocarcinomas in the Western world^2^, there are still some uncertainties and disagreement about the staging and the treatment of this disease. In the past, GEJ adenocarcinomas were staged either as an oesophageal or gastric cancers, depending on the centre of the tumour. According to the UICC 7^th^ criteria, they are now classified along oesophageal adenocarcinom^3^, although some investigators still consider them to be stomach carcinomas. Most of us agree that these tumours should be treated separately from other tumours of stomach and oesophagus, because they differ in terms of epidemiology, pathogenesis, surgical approach and in prognosis, as well. Based on the anatomic location of the tumour centre, GEJ adenocarcinomas are subclassified by Siewert into three types: type I are tumours of distal oesophagus; type II tumours (also termed true carcinomas of the cardia) arise immediately at the gastroesophageal junction; type III tumours have subcardial centre with predominant involvement of the proximal stomach or gastric cardia.^1^

The principal treatment of nonmetastatic GEJ adenocarcinomas is the surgical resection like in others gastrointestinal tumours.^4^–^6^ There exist several different surgical approaches, depending on the localization of tumour, but the common goal is en-bloc removal of the entire tumour with adequate lymphadenectomy. The locoregional disease recurrence is observed in 25–80% of patients operated on.^7^,^8^ A number of studies were carried out in order to try to improve the survival of the patients with GEJ adenocarcinomas. Uncertainties still remain, because GEJ patients were included either in gastric or oesophageal cancer studies.^9^–^14^ Therefore, the optimal multimodal treatment strategy is still to be determined. However, it is clear that the patients with T2, T3 and/or N+ disease need additional treatment to surgery alone.

Preoperative chemotherapy with epirubicine (E), cisplatin (C) and fluorouracile (FU), or CFU regimen improves the overall survival in some, but not in all trials.^9^,^15^–^17^ Preoperative radiochemotherapy trials gave mixed results^10^,^11^, but meta-analysis comparing preoperative chemotherapy with chemoradiotherapy confirmed that the trimodal therapy has higher 2-years overall survival rate (13% *vs.* 7%).^18^

Randomized studies of adjuvant radiotherapy only did not report any benefit.^19^,^20^ Benefit of adjuvant chemotherapy only is also questionable.^12^,^21^,^22^ However, Intergroup 0116, a randomized phase III trial of adjuvant radiochemotherapy, showed benefit for adjuvant radiochemotherapy compared to observation only after the surgery. In the study, 556 patients with resected gastric cancer were included, 20% of them with the adenocarcinomas of GEJ. Higher 3-years disease free survival (48% *vs*. 31%; p=0.001) and 3-years overall survival (50% *vs*. 41%; p=0.005) were observed for patients treated with surgery and adjuvant radiochemotherapy compared to those treated with surgery only. Critic of this trial was the lack of optimal lymphadenectomy, since only 10% of patients had D2 lymphadenectomy and only 36% had D1 lymphadenectomy. The rest of patients underwent D0 lymphadenectomy. It has been postulated that adjuvant radiochemotherapy compensated for suboptimal surgical procedures thus resulting in an overestimation of the survival benefit.^13^ Despite these doubts there has also been proved benefit of adjuvant chemoradiotherapy after D2 lymphadenectomy in the study of Kim *et al.* with 544 patients.^14^

However, no comparative data of all these multimodal approaches have been published so far.

In the Institute of Oncology Ljubljana, nowadays preoperative chemoradiotherapy is the treatment of choice. There are still some patients with locoregionally advanced disease in whom the surgical resection is performed as the first treatment, followed by postoperative radiochemotherapy. Some of these patients have tumours clinically staged as T1–2 N0, but at the time of the surgery more advanced disease is determined. Some other patients have advanced but technically resectable disease with profuse bleeding or other conditions that require an immediate surgical intervention.

The main endpoints of this retrospective study were to find locoregional control (LRC), disease-free survival (DFS), disease-specific survival (DSS), and overall survival (OS) in patients with resectable GEJ adenocarcinoma, who were treated with postoperative radiochemotherapy in Slovenia in the period 2005–2010.

## Patients and methods

### Patients and tumour characteristics

In the period from January 2005 to June 2010, 70 patients (55 males and 15 females; aged 34–77 years, mean age 60 years) were treated for nonmetastatic adenocarcinoma of GEJ with postoperative concomitant chemoradiation at the Institute of Oncology Ljubljana, Slovenia. As the Institute of Oncology is the only hospital in Slovenia with radiotherapy facilities, this number represents the total population of patients with operable GEJ adenocarcinomas treated with adjuvant radiochemotherapy in the country. All patients had locally or regionally advanced disease without distant metastases (stages IIa–IIIc) (Table 1).

### Surgical treatment

Of 70 patients, 63 (90%) were operated on in two major surgical centres in Slovenia, at the University Medical Centres in Maribor and Ljubljana, and the remaining 7 (10%) patients in one of the Slovenian regional hospitals. Proximal subtotal resection of the stomach was performed in 3 patients (4.3%), total resection of the stomach in 48 patients (68.5%), transhiatal esophagogastrectomy in 14 patients (20%), and transthoracal esophagogastrectomy in 5 patients (7.1%). As determined on the histopathological examination of surgical specimen, the radical resection (R0) was performed in 56 (80%) patients and in the remaining 14 (20%) patients non-radical surgery was performed - R1 resection in 11 patients (15.7 %) and R2 in 3 patients (4.2%).

### Tumour characteristics

Most frequently (in 32 patients; 45.7%), the primary tumour originated in the subcardial stomach and infiltrated the GEJ (Siewert III). In 10 patients (14.3%) tumour originated in the distal oesophagus (Siewert I) and in 14 patients (20%) in the cardia (Siewert II). In 14 patients (20%) the tumours extended over large area and for this reason their classification was not possible. The tumour was staged as pT2 in 23 patients (32.8%), pT3 in 41 patients (58.6%) and as pT4 in 6 patients (8.6%). Sixty-five patients (92.9%) had N+ disease (Table 1).

### Investigations before and during therapy

After the surgery, all patients with the disease of pathological stage II or higher, were presented to a multidisciplinary advisory team, consisting of a surgeon, radiation oncologist and medical oncologist, in order to assess the prospects of the eventual adjuvant treatment. All patients underwent a general clinical examination and blood counts. The patients with heart, liver or renal diseases and those with poor performance status (≥2 according to the World Health Organization - WHO) were assessed as non-eligible for the adjuvant therapy. The investigations performed before the surgery to define the extent of the disease and to rule out metastatic disease, such as esophagogastroduodenoscopy, endoscopic ultrasound (EUS) of tumour areas, computer tomography (CT) of the thorax or abdomen, and PET-CT, were repeated only in the patients in whom the progression of the disease was clinically suspected.

During the therapy, the patients were clinically examined and referred to haematology and biochemistry blood tests once a week. The therapy-related local and systemic toxicity was assessed according to National Cancer Institute Common Toxicity Criteria (NCI-CTC) version 2.0.^23^ The performance status of patients was determined and their body weight was measured on the weekly basis. During the treatment all patients were monitored by the nutritionist as well.

### Postoperative radiochemotherapy

The adjuvant treatment was initiated six to eight weeks after the surgery. The treatment schedule included 6 cycles of chemotherapy with 5-FU (1000 mg/m^2^) in 96 hours continuous infusion and cisplatin (75 mg/m^2^) in a bolus on day 2 of each cycle. The treatment cycle was repeated every 28 days. Chemotherapy administration required hospitalization for appropriate monitoring, hydration, antiemetic therapy and other supportive treatment. Radiotherapy was supposed to start at the beginning of the second cycle. Three-dimensional conformal radiotherapy was performed using 15 MV photon beam linear accelerator. A prescribed dose was 45 Gy with daily fraction of 1.8 Gy, five times per week. The dose specification was based on the International Commission on Radiation Units (ICRU) Report 50 recommendations.^21^ Treatment field borders were based on pretreatment investigations and imaging and postoperative anatomy, with tumour bed and regional lymph node areas included. Dose-volume histograms were checked to verify that radiation plans were optimized regarding target coverage and normal tissue sparing. The position of individual irradiation fields was checked on the weekly basis.

In case of severe therapy-related toxicity, irradiation and/or chemotherapy doses were modified and adapted to the patient’s physical condition or laboratory tests. When necessary, chemotherapy application was delayed, or radiotherapy was temporarily interrupted or terminated.

### Follow-up

After the completion of the treatment, patients performed regular follow-up visits. Physical examination and routine laboratory tests with tumour markers CEA, Ca 19-9 and Ca 72-4 were made every three months for the first two years after the treatment, every sixth months between two and five years after the treatment and thereafter once a year. Imaging investigations, CT of the thorax and abdomen and/or US of abdomen and chest X-ray, were performed two times per year for the first two years of the follow-up and then once a year. In case of suspected recurrence of the disease, other investigations such as endoscopy, EUS, magnetic resonance imaging of suspected area and PET-CT, were also performed.

### Statistical analysis and ethical consideration

The statistical analysis was performed using personal computer and software statistical package SPSS, version 18 (SPSS Inc., USA). LRC was defined as the period from the date of surgery till the local and/or regional recurrence, DFS till the local, regional or systemic recurrence, DSS till the death due to GEJ adenocarcinoma, and OS till the death from any cause, respectively. The survival of patients was computed from the date of surgery to the September 15, 2011 (close-out date). The survival probability was calculated using Kaplan-Meier estimate^25^, and the log rank test was used to evaluate the differences between individual groups of patients.^26^ Independent prognostic values of variables that appeared as statistically significant on univariate analysis were tested by multivariate Cox regression analysis model.^27^ Two-sided tests were used and the differences at p < 0.05 were considered as statistically significant.

The retrospective study was carried out according to the Declaration of Helsinki.

## Results

### Outcome of disease

The median follow-up time of all 70 patients was 17.7 months (range: 3.3–64 months), whereas in survivors it was 27 months (range: 3.7–64 months). On the close-out date, 27 (38.6%) patients were still alive, 20 (28.6%) of them being with no signs of disease. Thirty-nine (55.7%) patients died from GEJ adenocarcinoma, and in 4 (5.7%) patients the cause of death could not be determined.

After adjuvant radiochemotherapy, the recurrence was observed in 46 (65.6%) patients. Local and/or regional recurrence developed in two (2.8%) patients at 20.3 and 33.4 months after the end of the treatment. Locoregional and systemic disease was observed in five (7.1%) patients in the median period of time of 11.4 months (range: 4.8–21 months), and distant metastases alone developed in 39 (55.7%) patients in the median period of time of 11.9 months (range: 3.3–36.5 months).

**FIGURE 1:**
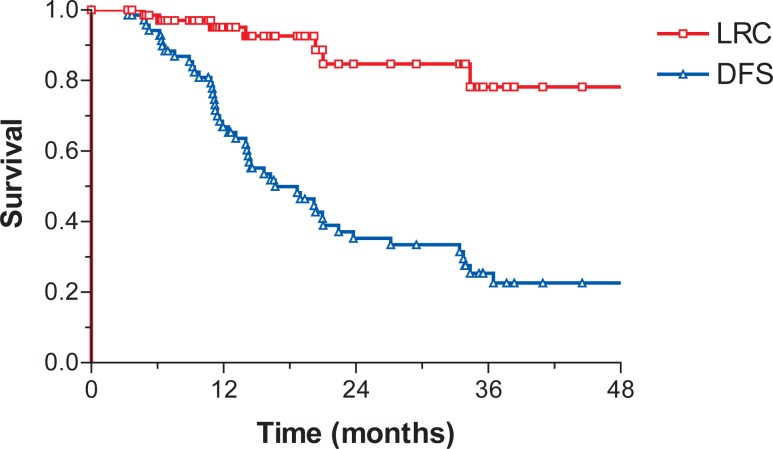
Locoregional control (LRC) and disease-free survival (DFS).

**FIGURE 2:**
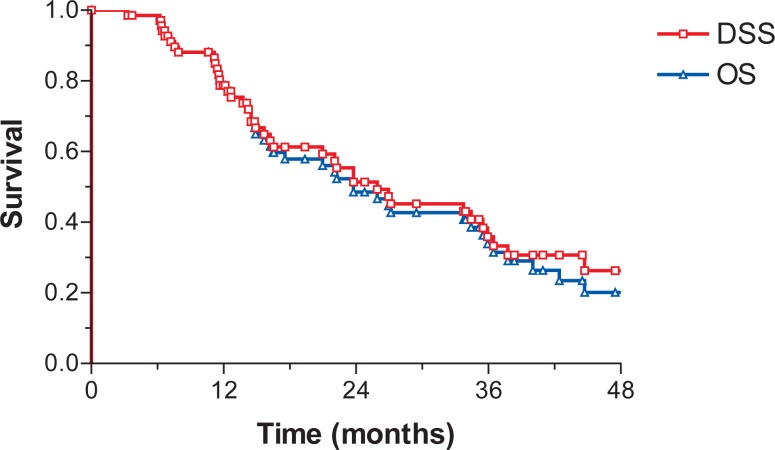
Disease-specific survival (DSS) and overall survival (OS).

Median LRC was not reached. Median DFS, DSS and OS were 16.7 months (95% C.I.: 10–23.4), 25.9 (95% C.I.: 13.7–38.2) and 23.8 months (95% C.I.: 17–30.5), respectively. At 3 years LRC, DFS, DSS and OS were 78.2%, 25.3%, 35.8%, and 33.9%, respectively (Figures 1 and 2).

### Toxicity of adjuvant radiochemotherapy

Postoperative chemotherapy started in the median time of 6 weeks (range: 4.4–10.7 weeks) after the surgery. The total postoperative treatment time ranged from 0.5 to 25.6 weeks (median 14.6 weeks), whereas the duration of the radiotherapy part of the protocol ranged from 1–5.6 weeks (median 5 weeks). Twenty-six patients (37.1%) completed the treatment according to the protocol. Sixty-seven patients (87.1%) reached the total radiation dose of 45 Gy, whereas in six patients (8.4%) the total delivered dose was lower (from 9–27 Gy). Three (4.3%) patients did not even start with the radiotherapy because of the side effects during the first cycle of chemotherapy. All six cycles of chemotherapy were administered in 26 patients (37.1%), 7 patients (10%) received five, 16 patients (22.9%) four, and 21 patients (30%) three cycles or less. No death occurred due to the therapy. Acute toxicity grade 3 or more, such as stomatitis, dysphagia, nausea and/or vomiting, and infection, occurred in 2.9%, 34.3%, 38.6% and 41.5% of patients, respectively (Table 2).

In 4 (5.7%) patients, an increase of body weight was recorded during the therapy, 5 (7.1%) patients maintained constant weight, whereas the remaining 61 (87.2%) patients lost their weight with respect to the weight they had at the beginning of the treatment. The maximum body weight loss was 20.5% (median 7.5%). Supplementary enteral nutrition was administered to 51 (72.9%) patients and for 20 (28.6%) patients parenteral nutrition was needed at least once during the treatment.

### Prognostic factors

In patients in whom splenectomy was performed as well, DFS (p=0.033), DSS (p=0.032) and OS (p=0.016) were lower. Patients with the higher number of involved lymph nodes (stage N2 or N3) had lower DFS (p=0.022) and OS (p=0.026). Patients with weight loss >5 kg before the operation had lower DSS (p=0.032) and OS (p=0.022). Patients with advanced disease (stage IIIb, IIIc) and patients with perineurial invasion had lower OS (p=0.025 and p=0.044, respectively). We did not find any differences in the survival regarding tumour localization classified by Siewert, tumour differentiation, type of the surgery and surgical specimen margins (R0 vs. R+). Older patients (aged 60 years or more) had higher DSS (p=0.045) and OS (p=0.053). Patients with levels of CEA more than 2 μg/L at the beginning of the postoperative treatment had lower DSS (p=0.023) and OS (p=0.028). Patients with levels of Ca 19-9 more than 20 kU/L at the beginning of the postoperative treatment had lower LRC, DFS, DSS and OS (p=0.018, p=0.001, p=0.007 and p=0.017, respectively).

In the multivariate analysis of the survival, splenectomy and level of Ca 19-9 >20 kU/L before the adjuvant treatment were identified as independent prognostic factors for the lower DFS (p=0.029 and p=0.004), DSS (p=0.012 and p=0.001) and OS (p=0.006 and p<0.0001). Age < 60 years, higher number of involved lymph nodes (stage N2 or N3) and advanced disease stage (stage IIIb or IIIc) were identified as independent prognostic factors for the lower DSS (p=0.009, p=0.019 and p=0.006, respectively) and OS (p=0.005, p=0.014 and p=0.003, respectively) (Table 3).

## Discussion

Patients with GEJ adenocarcinomas treated with surgery only have a very poor prognosis with a 5-year survival rate of approximately 20%.^28^,^29^ Many authors proved that the combined radiotherapy and chemotherapy, applied preoperatively, postoperatively or at inoperable patients might significantly improve the survival of these patients.^10^,^11^,^13^,^14^,^18^,^30^ In our study the retrospective analysis of 70 patients with GEJ adenocarcinomas treated with postoperative radiochemotherapy was performed. The radical resection was performed in 80% of our patients, which could be comparable to results of other published studies.^31^–^34^ In our study the 3-year LRC, DFS, DSS and OS results should not be compared with results of MacDonald *et al.*^13^, because the majority of patients in his study had stomach carcinoma and only 20% patients had GEJ carcinoma. The subanalysis for those patients was not performed. The other reason why the data are not comparable is the use of different cytostatics; in MacDonald′s study chemotherapy included 5-FU and leucovorin and in our study 5-FU and cisplatin were administered. In MacDonald′s study, patients treated with postoperative radiochemotherpy had 48% 3-years DFS and 50% 3-years OS. The study of Adelstein *et al*.^31^ in which 50 patients with carcinomas of oesophagus and GEJ treated with postoperative radiochemotherapy with 5-FU and cisplatin were included, reported that 4-year LRC, DFS and OS were 86%, 50% and 51%. These results are better than ours, but in their study also patients with less advanced disease and, therefore, a better prognosis were included.

Although one of the critique of the American intergroup study^13^ was referred to the high percentage of patients (36%), who did not complete the regimen, in our study only 37% finished the therapy according to the protocol. While most of our patients received full radiation therapy (87.1%), all six cycles of chemotherapy were applied in only 26 patients (37.1%). The most common toxic side effects classified as grade 3 or higher were, as in MacDonald’s study^13^, gastrointestinal toxic effects, infections and leucopenia. In Adelstein′s study^31^ only 3 patients did not finished the treatment according to the protocol, but their schedule contained only 2 cycles while our schedule contained 6 cycles of chemotherapy with 5-FU and cisplatin. Therefore, greater toxicity and more treatment interruptions in our study were expected.

Our analysis demonstrated that patients with the involvement of numerous lymph nodes and more advanced stage of disease have a lower survival. These are well known factors that have an impact on the survival of patients with GEJ carcinoma.^35^–^41^

Tumour origin (tumour localization classified by Siewert), tumour differentiation, type of surgery and positive surgical margins did not have any impact on the outcome as in some other series as well.^31^,^42^ On the other hand, some other authors reported that patients with GEJ adenocarcinomas Siewert type I have a better prognosis because they have an earlier onset of symptoms (like dysphagia) and are, therefore, diagnosed in earlier stages. They also argued that poor differentiation of tumours has negative effect on the survival due to the increased risk of lymphatic dissemination and that the presence of tumour cells in the resected margins could have a negative impact on the survival of operated patients.^35^

Weight loss is a common symptom of GEJ cancer. It is due to the mechanical effects of the tumour that causes dysphagia, early satiety, nausea and vomiting. Beside this, systemic influences like hypermetabolism, anorexia and altered protein metabolism, have an important role in the nutritional status of these patients.^43^ In our study, weight loss of more than 5 kg before the operation was associated with the lower DFS, DSS and OS. In several other studies – not only at patients with GEJ adenocarcinoma – weight loss before the treatment was a negative prognostic factor.^42^,^44^–^47^ In a large study of patients with oesophageal cancer, weight loss greater than 10% of pre-morbid weight was the only significant predictor of early death in patients undergoing the surgical resection.^45^

Our analysis demonstrated the survival benefit for patients older than 60 years. Similar results were described in the study of Crumley *et al.* who found that patients older than 65 years had better 3-year survival than younger patients (32% vs. 29%; p=0.017).^42^ Some other studies did not find any prognostic significance between different groups of age.^48^–^51^

The multivariate analysis also identified splenectomy as a negative prognostic factor. In several published studies splenectomy had a negative impact on the survival,^52^–^53^ while other studies did not demonstrate marked effect on the survival.^33^,^34^,^54^ The need for splenectomy in patients with GEJ is still controversial. Compared with a gastric cancer, in GEJ cancer lymph node metastases in the splenic hilum are more frequent and that is why some surgeons consider splenectomy as necessary.^55^,^56^ However, splenectomy is known to be associated with increased morbidity after the resection of proximal gastric and GEJ cancer, especially due to a higher risk of infections.

One of the independent prognostic factors in our study was also the level of Ca 19-9 more than 20 kU/L before the start of the postoperative treatment. Elevated tumour markers are known to be associated with the higher probability of lymph node metastases, lymphatic and blood vessel invasion, depth of invasion, higher stage and dissemination of the disease. Kočevar *et al.*^57^ reported that translationally controlled tumour protein (TPT1) was shown to be differentially expressed only in patients GEJ cancer, but, it clinical have to be established.

## Conclusions

Postoperative radiochemotherapy for GEJ is an attractive approach for several reasons since the treatment decision is based on the true pathologic stage and hence a more accurate assessment of the disease extent. On the other hand, it also has some disadvantages, such as difficult recovery of some patients after extensive resections and worse blood supply and oxygenation of tumour bed and, therefore, less effective treatment with radiochemotherapy. However, even if we consider that preoperative radiochemotherapy can improve resectability and, therefore, enables us the higher proportion of curative resections, postoperative radiochemotherapy is still reserved for the selected group of patients, who first underwent surgery due to different reasons.

## Figures and Tables

**TABLE 1:** Patients and tumour characteristics

**Characteristics**		**No.**	**%**
Gender	Male	55	78.6
	Female	15	21.4
Tumour classification by Siewert	Type I	10	14.3
	Type II	14	20
	Type III	32	45.7
	Undetermined	14	20
pT – stage	1	0	0
	2	23	32.9
	3	41	58.5
	4	6	8.6
pN – stage	0	5	7.1
	1	26	37.1
	2	25	35.7
	3	14	20
Overall stage	IIa	4	5.7
	IIb	11	15.7
	IIIa	25	35.7
	IIIb	13	18.6
	IIIc	17	24.3
Tumour differentiation	Well	5	7.1
	Moderately	20	28.6
	Poor	39	55.7
	Unknown	6	8.6
Surgical procedures	Transhiatal oesophagogastrectomy	14	20
	Transthoracic oesophagogastrectomy	5	71
	Proximal subtotal gastrectomy	3	4.3
	Total gastrectomy	48	68.5
Surgical margins	Negative	56	80
	Positive	14	20
Perineurial invasion	Yes	39	55.7
	No	16	22.9
	Unknown	15	21.4
Lymphovascular invasion	Yes	40	57.1
	No	6	8.6
	Unknown	24	34.3
Angioinvasion	Yes	20	28.6
	No	23	32.9
	Unknown	27	38.6

pT = pathological T-stage; pN = pathological N-stage

**TABLE 2:** Toxicity of adjuvant radiochemotherapy

**Toxicity**	**NCI grade (%)**					
	**0**	**1**	**2**	**3**	**4**	**total**
Stomatitis	87.1	5.7	4.3	2.9	0	100

Radiodermatitis	97.1	1.4	1.4	0	0	100
Diarrhoea	74.3	21.4	4.3	0	0	100
Dysphagia	31.4	18.6	15.7	34.3	0	100
Nausea, vomiting	14.3	28.6	18.6	38.6	0	100
Infection	28.6	5.7	24.3	38.6	2.9	100
Leucocyte count	17.1	15.7	35.7	27.1	4.3	100
Haemoglobin level	8.6	57.1	31.4	2.9	0	100
Platelet count	60	28.6	7.1	2.9	1.4	100

**TABLE 3:** Multivariate analysis of survival

**Prognostic factors**	**n**	**Locoregional control**	**Disease free survival**	**Disease specific survival**	**Overall survival**

		**p**	**p**	**p**	**p**
*pT- stage*					
pT 1+2	48				
pT 3+4	75			0.03	
*pN- stage*					
pN 0+1+2	98				
pN 3	25				
*Overall stage*					
Stage Ib –III	93				
Stage IV	30	0.05			
*Stomach involvement*					
Whole stomach	7				
Individual areas	116				
*Primary tumour site*					
Cardia	16				
Other sites	107				
*Perineurial invasion*					
Yes	45				
No	59				
*Angioinvasion*					
Yes	45		0.07		
No	23				
*Initial Hb level*					
Hb start ≤ 110 g/l	24				
Hb start > 110 g/l	99	0.009	0.0001	0.02	0.01
*5-FU total dose per cycle*					
≤ 4000 mg	109				
> 4000 mg	14		0.03	0.07	

pT = pathological T-stage; pN = pathological N-stage; Hb start = haemoglobin concentration at the start of the therapy
